# Soy intake and breast cancer risk in Singapore Chinese Health Study

**DOI:** 10.1038/sj.bjc.6604448

**Published:** 2008-07-01

**Authors:** A H Wu, W-P Koh, R Wang, H-P Lee, M C Yu

**Affiliations:** 1 Department of Preventive Medicine; University of Southern California Keck School of Medicine, Los Angeles, CA 90089, USA; 2 Department of Community, Occupational and Family Medicine, Yong Loo Lin School of Medicine, National University of Singapore, Singapore; 3 The Cancer Center, University of Minnesota, Minneapolis, MN 55455, USA

**Keywords:** soy, menopausal status, body size, hormone receptor, breast cancer, Chinese

## Abstract

We investigated the effects of soy isoflavone intake on breast cancer in a prospective study of 35 303 Singapore Chinese women enrolled during April 1993 to December 1998 in the Singapore Chinese Health Study. At recruitment, each subject was personally administered a validated semiquantitative food frequency questionnaire covering 165 food and beverage items. As of December 31 2005, 629 had developed breast cancer following an accumulation of 338 242 person-years. Using Cox regression and adjusting for age at interview, year of interview, dialect group, education, family history of breast cancer, age when periods became regular, parity, menopausal status, body mass index (BMI), n-3 fatty acid, and other covariates, we found breast cancer risk was reduced significantly in association with high soy intake. Relative to women with lower (below median) soy intake (<10.6 mg isoflavone per 1000 Kcal), women with higher (above median) intake showed a significant 18% risk reduction (relative risk (RR)=0.82, 95% confidence interval (CI)=0.70–0.97). This inverse association was apparent mainly in postmenopausal women (RR=0.74, 95% CI=0.61–0.90), and was not observed in premenopausal women (RR=1.04, 95% CI=0.77–1. 40). Among postmenopausal women, the soy–breast cancer association was stronger in those above median BMI (RR=0.67, 95% CI=0.51–0.88) than in leaner women (RR=0.83, 95% CI=0.62–1.11). Duration of follow-up modified the soy–breast cancer association, the effect being twice as large among women with 10+ *vs* fewer years of follow-up. Neither oestrogen nor progesterone receptor status of the tumours materially influenced the association. These prospective findings suggest that approximately 10 mg of isoflavones per day, obtained in a standard serving of tofu, may have lasting beneficial effects against breast cancer development.

Of at least 28 detailed studies of soy and breast cancer risk published in English since 1990, 14 were in western populations with very low intake of soy (<1 mg of isoflavone per day) and in these, intake was unrelated to breast cancer risk (Wu *et al*, 2008). Of the other 14 studies in Asia or in Asian–Americans with substantially higher soy intake, eight (Lee *et al*, 1991; Dai *et al*, 2001; Yamamoto *et al*, 2003; Hirose *et al*, 2005; Lee *et al*, 2005; Shannon *et al*, 2005; Do *et al*, 2007) covered the main sources of soy intake and carefully adjusted for relevant potential confounders. In a meta-analysis of these eight studies, we found a stepwise reduction in breast cancer risk with increasing soy intake. Compared to the lowest soy intake (<5 mg isoflavones per day), risk of breast cancer reduced significantly by 12% in association with moderate intake (∼10 mg isoflavones per day) and 29% in association with high intake (20 isoflavones or more per day; Wu *et al*, 2008). However, only one Asian study was of cohort type (Yamamoto *et al*, 2003). Assessment of soy intake was incomplete in two other cohort studies (Key *et al*, 1999; Nishio *et al*, 2007) and they were not included in our meta-analysis. Since the publication of our review on soy and breast cancer, no association with soy intake was found in a British cohort with moderate soy intake (Travis *et al*, 2008). Thus, additional cohort studies of soy and breast cancer risk are needed in populations with substantial soy intake.

We have, therefore, investigated soy intake and breast cancer risk in the Singapore Chinese Health Study (SCHS), a cohort study of diet and cancer.

## Materials and methods

The SCHS was a prospective investigation of the role of dietary factors in cancer aetiology (Hankin *et al*, 2001). Briefly, 35 303 Chinese women and 27 954 Chinese men aged 45–74 years of Hokkien or Cantonese dialect group were enrolled in the population-based cohort study between April 1993 and December 1998. At recruitment, information on lifestyle factors, usual diet, and reproductive history (for women only) was obtained through in-person interviews. The food frequency questionnaire (FFQ) covered usual intake during the previous 12 months of 165 items identified in a prior 24-h recall study to represent the major food and beverage items in this population. Frequency of consumption was assessed using eight categories for foods and nine categories for beverages, ranging from ‘never or hardly ever’ to ‘two or more times per day.’ Colour photographs of portion sizes (small, medium, and large) were used to aid with recall of amounts consumed. A food composition table was developed, and items on the questionnaire were linked to the food composition database.

There are seven common soy products in the Singapore Chinese diet and all are nonfermented. We expressed total soy intake in terms of grams of soy protein, soy isoflavones, and equivalent amounts of tofu per day (Wu *et al*, 2002). Soy protein intake was calculated using the Singapore Food Composition Table (Hankin *et al*, 2001). Total soy isoflavone intake was estimated from the summation of the genistein, daidzein, and glycitein contents that have previously been measured in market samples of common soy foods in Singapore. Equivalent amounts of tofu per day were calculated to facilitate comparison with a known dietary item while taking into account the varying water contents across the seven soy foods. This was carried out as follows: the water content of the various soy-containing foods varies from 54% for cooked foojook, 58% for cooked foopei, 69% for cooked taukwa, and 89% for cooked plain tofu to 92% for soybean drink. We, therefore, determined that 1 **g** of cooked foojook is equivalent to 4.2 **g** (46/11) of plain tofu, 1 **g** of cooked foopei is equivalent to 3.8 **g** (42/11) plain tofu, and so on. The total soy intake for each subject was estimated as the summation of all soy foods expressed in units of plain tofu equivalent. In a dietary validation study of SCHS subjects, we found correlation coefficients of 0.63, 0.39, and 0.32 between the FFQ and a series of 24-h recalls for tofu, isoflavone, and soy protein, respectively. Furthermore, dietary intake of soy was statistically significantly correlated with urinary isoflavonoid (Seow *et al*, 1998).

The SCHS has been continuously followed, both actively through re-interviews and passively through computer linkage to population-based death and cancer registries in Singapore (Seow *et al*, 1996). The nationwide cancer registry has been in place since 1968 and is complete in its recording of cancer (Parkin *et al*, 2002). Loss to follow-up in the cohort has been negligible.

As of 31 December 2005, 629 women had developed breast cancer among the 34 028 participants following an accumulation of 338 242 person-years (1275 of the 35 303 women with prevalent cancer at baseline were excluded). Histological, staging information, oestrogen receptor (ER), and progesterone receptor (PR) status on all breast cancer diagnoses were confirmed by manual review of the pathology reports and clinical charts.

### Statistical analysis

Data were analysed using standard methods for cohort studies. We calculated person-years from the baseline questionnaire to the date of breast cancer diagnosis, death or December 31, 2005, whichever was sooner. Proportional hazards regression methods were used to examine the association between quartiles of isoflavone intake and breast cancer risk, measured by relative risks (RR) and their corresponding 95 percent confidence intervals (95% CI) in all subjects and among those postmenopausal at baseline. Relevant demographic factors including age (years) at recruitment, year of recruitment (1993–1998), dialect group (Cantonese, Hokkien), education (no formal education, primary school, secondary school, or higher), and established risk factors including age when period became regular (<12, 13–14, 15–16, and 17+ years or period never became regular), number of live births (none, 1–2, 3–4, and 5+), body mass index (BMI; continuous, kg m^−2^), menopausal status, and n-3 fatty acid intake were adjusted in the analysis. As adjusting for green tea intake, tobacco smoking (never and ever), alcohol intake (nondrinker, monthly, weekly, and daily), and history of diabetes left the results unchanged; we show results without these further adjustments. We conducted subgroup analyses of soy isoflavone intake and risk, stratified by menopausal status and examined the potential effect modification by including interaction terms for body size. We also investigated the effect of soy isoflavone intake by ER and PR status (positive, negative, and unknown), and ER/PR status combined (positive/positive, negative/negative, and unknown/unknown). *P*-value less than 5% are considered statistically significant and all *P*-values quoted are two-sided.

## Results

The mean age of breast cancer patients at diagnosis was 62.0 years (standard deviation (s.d.) 8.2 years; range: 46–86 years). The mean interval between interview and diagnosis was 5.6 years (range: 1 month–12 years). Table 1 shows the association between intake of soy isoflavone and risk. Risk was significantly reduced (adjusted RR=0.82, 95% CI=0.70–0.97; *P*=0.019) among women with above median soy intake (>10.6 mg 1000 Kcal^−1^) compared to those with lower intake (<=10.6 mg 1000 Kcal^−1^). Results were unchanged after adjustment for intake of green tea, tobacco smoking, alcohol intake, and history of diabetes (data not shown). Table 1 also shows the associations stratified by menopausal status at enrollment. Soy intake was unrelated to risk in premenopausal women (RR=1.04, *P*=0.82), but was significantly inversely associated with risk in postmenopausal women with an adjusted RR for those above the median of 0.74 (95% CI=0.61–0.90; *P* for interaction=0.078).

The inverse association between soy intake and breast cancer was statistically significant in postmenopausal women with higher (above median value for all women in the cohort) BMI (adjusted RR=0.67, 95% CI=0.51–0.88) but not in those below the median BMI value (adjusted RR=0.83, 95% CI=0.62–1.11) (Table 2). The two sets of RRs were not statistically different from each other (*P* for interaction=0.29). Table 3 shows that there were no material differences in the relationship with soy by hormone receptor status in postmenopausal women.

The protective effect of high soy intake was very clear and statistically significant among women with 10 or more years of follow-up (RR=0.48, 95% CI=0.29–0.78) (Table 4). High soy intake was less strongly associated with risk reduction among women with less than 10 years of follow-up (RR=0.88, 95% CI=0.74–1.05). Results were similar when we restricted the analysis to postmenopausal women (Table 4).

## Discussion

This study found a significant 18% reduction in breast cancer risk in association with the above median soy isoflavone intake (⩾10.6 mg 1000 Kcal^−1^), a degree of risk reduction compatible with the results that we found in a combined analysis of eight studies in Asian populations with soy intake (Wu *et al*, 2008). The amount of soy isoflavones (∼10 mg per day) implicated to have benefit in cancer prevention can be obtained in one standard serving of tofu (Trock *et al*, 2006). The inverse association with soy intake in this Singapore cohort was statistically significant in postmenopausal but not in premenopausal women (Table 1), but we were limited in studying such women by the lower age limit of this cohort study being 45 years, and only 26% of participants were aged 45–50 years at enrollment. There is a suggestion that heavier postmenopausal women showed a stronger association than leaner women, although the two sets of RRs are not statistically different from each other (Table 2). The risk reduction with soy intake was found in both ER+ and ER− tumours as well as in PR+ and PR− tumours (Table 3). A stronger soy–breast cancer association was found among women with longer (10+ years) duration of follow-up (Table 4).

To date, eight cohort/nested case–control studies have investigated the association between soy intake and breast cancer risk, of which four were not very informative, being conducted in western populations with very low soy intake (average of ∼1 mg of soy isoflavones per day). Of these, no association was found in three studies in populations with very low soy intake (Horn-Ross *et al*, 2002; Keinan-Boker *et al*, 2004; Touillaud *et al*, 2006), whereas one study found an increased risk (Grace *et al*, 2004).

The other four cohort studies were in populations with moderate-to-high soy intake, one in the Oxford arm of the European Prospective Investigation into Cancer and Nutrition (EPIC-Oxford), one of the few non-Asian populations with moderate soy intake. The mean daily soy isoflavone intake was 2.9 mg among non-vegetarians and 10.2 mg among vegetarians, but only about 30% of the 37 643 cohort participants were vegetarians, limiting the investigation of this question (Travis *et al*, 2008). Three studies were conducted in Japan (Key *et al*, 1999; Yamamoto *et al*, 2003; Nishio *et al*, 2007) with high soy intake. Our finding of a significant inverse association between soy intake and breast cancer, particularly in postmenopausal women is similar to that reported in the Japan Public Health Center-Based Prospective Study (JPHC) (Yamamoto *et al*, 2003). Soy intake in the JPHC study was obtained using a validated FFQ including intake of five soy foods, covering approximately 90% of the total soy isoflavone intake in Japan (Yamamoto *et al*, 2001). In contrast, breast cancer risk was unrelated to soy intake in two other Japanese cohort studies (Key *et al*, 1999; Nishio *et al*, 2007); assessment of soy intake was less complete in both studies and considerable random misclassification of soy exposure is likely to have led to underestimation of risk (ie, bias towards the null).

After the menopause, adipose tissue is the major site for oestrogen synthesis and high body size is associated with the elevated endogenous oestrogen (Judd *et al*, 1982). We noted a stronger association among postmenopausal women with below *vs* above median BMI, although the two sets of RRs were not statistically different from each other. In a case–control study among women in Shanghai, China, the inverse association was more evident in women with higher BMI (25+ kg m^−2^) than women with lower BMI irrespective of menopausal status (Dai *et al*, 2001). In the four cohort studies reviewed in the previous paragraph, the soy–breast cancer association was not examined separately by BMI category.

Our results show comparable effects of soy on ER+ and ER− tumours as well as PR+ and PR− tumours (Table 3). Of the eight studies included in our meta-analysis on soy and breast cancer in Asian populations (Wu *et al*, 2008), only one study in our meta-analysis investigated the risk by hormone receptor status, finding the effect more apparent for tumours that were positive for both ER and PR (Dai *et al* (2001). Further investigation of the soy–breast cancer association by menopausal status and ER/PR status will be needed.

Three studies (Shu *et al*, 2001; Wu *et al*, 2002; Thanos *et al*, 2006), including a case–control study that we conducted among Asian American women in Los Angeles County, found that the timing of soy exposure is an important codeterminant of risk, with stronger effects from exposures at earlier ages. This study lends further support to soy exposures occurring during the earlier stages of carcinogenesis and would result in a greater degree of reduction in a woman's subsequent risk for breast cancer. We noted that the magnitude of risk reduction among women with 10 or more years between exposure assessment and cancer diagnosis was twice than that among their counterparts with shorter time intervals. Interestingly, this model may explain the seemingly contradictory findings from recent soy intervention studies with generally null findings (Maskarinec *et al*, 2004; Wu *et al*, 2005).

This prospective study has several important strengths. First, information on soy intake and other risk factors was obtained in-person before cancer diagnosis and thus the recall bias is not a concern. This is particularly important as seven of the eight studies in our meta-analysis on soy and breast cancer were case–control studies (Wu *et al*, 2008). Second, we used a validated FFQ, our assessment of soy intake was relatively complete, and dietary intake of soy was statistically significantly correlated with urine isoflavone levels (Seow *et al*, 1998). Third, our study is larger than previous cohort studies on this topic (two of the previous studies had less than 200 breast cancer cases (Yamamoto *et al*, 2003; Nishio *et al*, 2007). The larger sample size, particularly of postmenopausal women, has allowed us to investigate the potential modifying effect of body size on the soy–breast cancer association in postmenopausal women. Finally, we were able to examine the risk pattern by hormone receptor status, apparently the first such from a prospective study.

In summary, among 34 028 women in the SCHS, high soy intake (⩾10.6 mg isoflavones per 1000 Kcal) was associated with a significant 18% reduction in breast cancer risk. Our results adds to the compelling results obtained from case–control studies on soy and breast cancer and suggest that the soy isoflavones may have lasting beneficial effects against breast cancer development. The level of soy isoflavones implicated to have benefit (∼10 mg isoflavones per day or one standard serving of tofu) is achievable even in populations that do not typically eat soy foods.

## Figures and Tables

**Table 1:** Intake of soy isoflavones and breast cancer risk

**Soy isoflavone (mg 1000 kcal^−1^)**	**Number of cases**	**Person-years**	**RR^a^**	**95% CI**
*All subjects*				
<10.6 mg	339	167 312	1.00	
⩾10.6 mg	290	170 930	0.82	0.70–0.97
*P*-value				0.019
				
*Menopausal status at baseline*				
*Premenopausal*				
<10.6 mg	84	43 668	1.00	
⩾10.6 mg	106	52 937	1.04	0.77–1.40
*P*-value				0.82
				
*Postmenopausal*				
<10.6 mg	255	123 608	1.00	
⩾10.6 mg	184	117 960	0.74	0.61–0.90
*P*-value				0.003
				
*P* interaction				0.08

a Adjusted for age, years of interview, dialect, education, family history of breast cancer, parity, age when period became regular, menopausal status (if applicable), body mass index, and n-3 fatty acid.

**Table 2:** Intake of soy isoflavones and breast cancer risk in postmenopausal women by body mass index

**Soy isoflavone (mg/1000 kcal)**	**Number of cases**	**Person-years**	**RR^a^**	**95% CI**
*BMI below median* (⩽*24* *kg* *m*^−*2*^)				
<10.6 mg	110	59587	1.00	
⩾10.6 mg	90	57772	0.83	0.62–1.11
*P*-value				0.21
				
*BMI above median* (>*24* *kg* *m*^−*2*^)				
<10.6 mg	145	64021	1.00	
⩾10.6 mg	94	60187	0.67	0.51–0.88
*P*-value				0.004
*P* interaction				0.29

a Adjusted for age, years of interview, dialect, education, family history of breast cancer, parity, age when period became regular, body mass index, and n-3 fatty acid.

**Table 3:** Intake of soy isoflavones and breast cancer risk (RR, 95% CI)^a^ in postmenopausal women by oestrogen receptor (ER) and progesterone receptor (PR) status

**Soy isoflavone (mg 1000 kcal^−1^)**	**Person-years**	**Hormone receptor (positive)**		**Hormone receptor (negative)**		**Hormone receptor (unknown)**	
		*N*	ER+	*N*	ER−	*N*	ER DK
<10.6 mg	122 868	111	1.00	56	1.00	88	1.00
⩾10.6 mg	117 370	73	0.67 (0.49–0.91)	36	0.66 (0.42–1.02)	75	0.89 (0.64–1.22)
*P*-value		0.01		0.06		0.46	
							
		*N*	PR+	*N*	PR−	*N*	PR DK
<10.6 mg	122 667	77	1.00	87	1.00	91	1.00
⩾10.6 mg	117 264	53	0.69 (0.48–1.00)	56	0.67 (0.47–0.95)	75	0.85 (0.62–1.17)
*P*-value		0.047		0.02		0.32	
							
		*N*	ER+PR+	*N*	ER−PR−	*N*	ER & PR DK^b^
<10.6 mg	122 651	74	1.00	51	1.00	88	1.00
⩾10.6 mg	117 262	52	0.70 (0.48–1.01)	35	0.70 (0.45–1.10)	75	0.89 (0.64–1.22)
*P*-value		0.060		0.12		0.46	
							

a Adjusted for age, years of interview, dialect, education, family history of breast cancer, parity, age when period became regular, body mass index, and n-3 fatty acid.

b We did not include breast cancers that were ER+ PR− (*n*=57), ER+PR unknown (*n*=1), ER−PR+ (*n*=4) or ER–PR unknown (*n*=2) in the analysis.

**Table 4:** Intake of soy isoflavones and breast cancer risk by the length of follow-up

**Follow-up (years)**	**Soy isoflavone (mg 1000 kcal^−1^)**	**All subjects (number of cases/person-years)**	**RR^a^ (95% CI)**	**Postmenopausal (number of cases/person-years)**	**RR^a^ (95% CI)**
0–<10 years					
	<10.6 mg	294/151 965	1.00	219/112 175	1.00
	⩾10.6 mg	262/154 053	0.88 (0.74–1.05)	166/105 940	0.81 (0.66–1.01)
			*P*=0.16		*P*=0.057
					
10+ years					
	<10.6 mg	45/15 347	1.00	36/11 434	1.00
	⩾10.6 mg	28/16 877	0.48 (0.29–0.78)	18/12 019	0.38 (0.21–0.68)
			*P*=0.003		*P*=0.001

a Adjusted for age, years of interview, dialect, education, family history of breast cancer, parity, age when period became regular, menopausal status (if applicable), body mass index, and n-3 fatty acid.
